# Serum Galectin-3 and Risk Stratification in Chronic Heart Failure: A Systematic Review of Mortality Outcomes

**DOI:** 10.7759/cureus.86959

**Published:** 2025-06-29

**Authors:** Saad Sulaiman, Razan Abdulhaleem Mohamed Khalafallah, Azza Elbadri, Tagwa Mohamed Abuelgasim Mohamedsalih, Alaa Mahmoud, Manahil Abdalla Mohamed Salih Abdelrahman, Ibrahim Mohammed Hassan

**Affiliations:** 1 Internal Medicine, Mid Cheshire Hospitals NHS Foundation Trust, Liverpool, GBR; 2 Clinical Hematology, Leicester Royal Infirmary, Leicester, GBR; 3 Acute Medicine, Worcestershire Royal Hospital, Worcester, GBR; 4 Pathology, Al Neelain University, Khartoum, SDN; 5 Cardiology, Lincoln County Hospital, Lincoln, GBR; 6 Internal Medicine, Russells Hall Hospital, Dudley, GBR; 7 Cardiology, Prince Sultan Cardiac Center, Najran, SAU

**Keywords:** biomarkers, chronic heart failure, galectin-3, mortality, prognosis, risk stratification

## Abstract

Galectin-3 (Gal-3) has emerged as a potential biomarker for risk stratification in chronic heart failure (HF), given its role in cardiac fibrosis and inflammation. However, its independent prognostic value and clinical utility remain controversial. This systematic review evaluates the association between serum Gal-3 levels and mortality outcomes in chronic HF patients, addressing its consistency, interaction with treatments, and comparative performance against established biomarkers. A systematic search was conducted in PubMed, Scopus, Web of Science, and Embase, adhering to the Preferred Reporting Items for Systematic Reviews and Meta-Analyses (PRISMA) 2020 guidelines. Twelve studies meeting the inclusion criteria (original research assessing Gal-3 and mortality in chronic HF) were selected. Data on study characteristics, prognostic value, and treatment interactions were extracted. Risk of bias was assessed using the Quality in Prognosis Studies (QUIPS) tool. Elevated Gal-3 levels were consistently associated with increased mortality in univariate analyses. However, its independent prognostic significance diminished in multivariate models adjusting for N-terminal pro B-type natriuretic peptide (NT-proBNP), soluble suppression of tumorigenicity-2 (ST2), or high-sensitivity troponin T (hs-TnT). Gal-3 demonstrated modifiability with therapies like sacubitril/valsartan but showed inconsistent interactions with beta-blockers (β-blockers) and mineralocorticoid receptor antagonists (MRAs). Compared to other biomarkers, Gal-3 underperformed in risk stratification, though it added value in multimarker panels. Methodological heterogeneity, particularly in assay standardization and cut-offs, was a key limitation across studies. While Gal-3 correlates with adverse outcomes in chronic HF, its incremental prognostic value is limited alongside established biomarkers. Standardization of measurements and further research into its therapeutic implications are needed. Gal-3 may currently serve best as part of a multimarker strategy rather than a standalone prognostic tool.

## Introduction and background

Chronic heart failure (HF) remains a leading cause of morbidity and mortality worldwide, with a progressive clinical course marked by frequent hospitalizations and poor long-term outcomes. Notably, HF in youth (<40 years) is a fast-growing epidemic across the globe, accounting for over 3% of all HF cases. Recent reports indicate that HF in younger populations is largely driven by the sharply rising prevalence of diabetes mellitus (DM), obesity, and metabolic syndrome, which confer increased inflammation at the myocardial level and resultant dysfunction [1]. Naturally, newer biomarkers, especially those related to inflammation and fibrosis, such as galectin-3 (Gal-3), are likely to offer great advantage in this group. Despite advances in pharmacological and device therapies, risk stratification in HF remains challenging, necessitating the identification of reliable biomarkers to improve prognostic accuracy and guide personalized treatment strategies. Gal-3, a β-galactoside-binding lectin, has emerged as a promising biomarker due to its involvement in cardiac fibrosis, inflammation, and adverse remodeling - key pathological processes underlying HF progression [2]. Elevated Gal-3 levels have been associated with worse clinical outcomes in HF patients, but its independent prognostic value and clinical utility relative to established biomarkers, such as natriuretic peptides and soluble suppression of tumorigenicity-2 (sST2), remain debated [3].

The pathophysiological role of Gal-3 in HF is linked to its profibrotic and proinflammatory actions [2]. Secreted by activated macrophages, Gal-3 promotes fibroblast proliferation and collagen deposition, contributing to myocardial stiffness and ventricular dysfunction [4]. Preclinical studies have demonstrated that Gal-3 inhibition attenuates cardiac fibrosis and improves function in animal models, suggesting its potential as a therapeutic target. Clinically, Gal-3 has been studied across diverse HF populations, including those with reduced or preserved ejection fraction, but inconsistencies in its predictive power persist [5]. While some studies report strong associations between Gal-3 and mortality, others suggest its prognostic significance diminishes when adjusted for confounding factors, such as renal function or comorbidities like diabetes [6]. This variability may stem from differences in study design, assay methods, or patient characteristics, underscoring the need for a systematic evaluation of the existing evidence.

Current HF guidelines emphasize the importance of multimarker approaches to refine risk prediction, yet the integration of Gal-3 into clinical practice has been limited by uncertainty about its incremental value [2]. Previous reviews have explored Gal-3 in HF, but none have systematically focused on its association with mortality outcomes while critically appraising methodological biases and interactions with treatments [7,8]. A comprehensive synthesis of the evidence is essential to clarify whether Gal-3 should be routinely measured for risk stratification or reserved for specific HF subgroups.

The aim of this systematic review is to evaluate the prognostic value of serum Gal-3 for mortality outcomes in patients with chronic HF, addressing three key questions: (1) How consistently is Gal-3 associated with all-cause and cardiovascular mortality in unadjusted and adjusted analyses? (2) Does Gal-3 modify or predict responses to HF therapies? (3) How does its prognostic performance compare to other biomarkers? By synthesizing evidence from observational and interventional studies, this review seeks to inform clinical practice and future research on the role of Gal-3 in HF risk stratification.

## Review

Methodology

Search Strategy and Data Sources

A systematic literature search was conducted in accordance with the Preferred Reporting Items for Systematic Reviews and Meta-Analyses (PRISMA) 2020 guidelines [9] to identify relevant studies evaluating the prognostic role of Gal-3 in chronic HF mortality outcomes. The search was restricted to four major electronic databases: PubMed, Scopus, Web of Science, and Embase. The search strategy included a combination of Medical Subject Headings (MeSH) terms and free-text keywords related to "galectin-3", "chronic heart failure", "mortality", "prognosis", and "risk stratification". No date restrictions were applied to ensure a comprehensive retrieval of relevant studies. The full search syntax was adapted for each database to account for differences in indexing and terminology (attached in Appendices).

Eligibility Criteria

Studies were included if they met the following criteria: (1) original research articles evaluating the association between serum Gal-3 levels and mortality outcomes in chronic HF patients; (2) prospective or retrospective cohort studies, randomized controlled trials (RCTs), or observational studies with clearly defined endpoints; (3) studies reporting hazard ratios (HRs), odds ratios (ORs), or other effect estimates with confidence intervals (CIs); and (4) studies published in English. Exclusion criteria included review articles, case reports, conference abstracts without full-text availability, and studies that did not assess mortality as a primary or secondary outcome.

Study Selection Process

All identified records from the database searches were imported into a reference management software, EndNote X9 (Thomson Reuters, Philadelphia, PA, USA), to remove duplicates. The remaining studies underwent a two-stage screening process. In the first stage, two independent reviewers screened titles and abstracts to exclude irrelevant studies. In the second stage, full-text articles of potentially eligible studies were assessed for final inclusion. Any discrepancies between reviewers were resolved through discussion or consultation with a third reviewer. The study selection process was documented in a PRISMA flow diagram, detailing the number of records identified, screened, excluded, and ultimately included.

Data Extraction

A standardized data extraction form was developed to collect relevant information from the included studies. Key extracted data included study characteristics, patient demographics, Gal-3 measurement methods, follow-up duration, mortality outcomes, and statistical findings. Data extraction was performed independently by two reviewers, and discrepancies were resolved through consensus.

Risk of Bias Assessment

The methodological quality and risk of bias of the included studies were assessed using the Quality In Prognosis Studies (QUIPS) tool [10], which evaluates six key domains: (1) study participation, (2) study attrition, (3) prognostic factor measurement (Gal-3), (4) outcome measurement, (5) adjustment for other prognostic factors, and (6) statistical analysis and reporting. Each domain was rated as low, moderate, or high risk of bias based on predefined criteria. Two reviewers independently performed the assessment, and any disagreements were resolved through discussion. The overall risk of bias for each study was summarized and presented in a table.

Data Synthesis and Analysis

Due to heterogeneity in study designs, patient populations, and Gal-3 measurement methods, a meta-analysis was not feasible. Instead, a narrative synthesis was conducted to summarize the prognostic value of Gal-3 across studies. Findings were categorized based on univariate and multivariate associations with mortality outcomes, interactions with treatment effects, and comparative performance with other biomarkers. Key trends and discrepancies were highlighted, and the clinical implications of the findings were discussed in the context of existing evidence.

Results

Studies Selection

**Figure 1:**
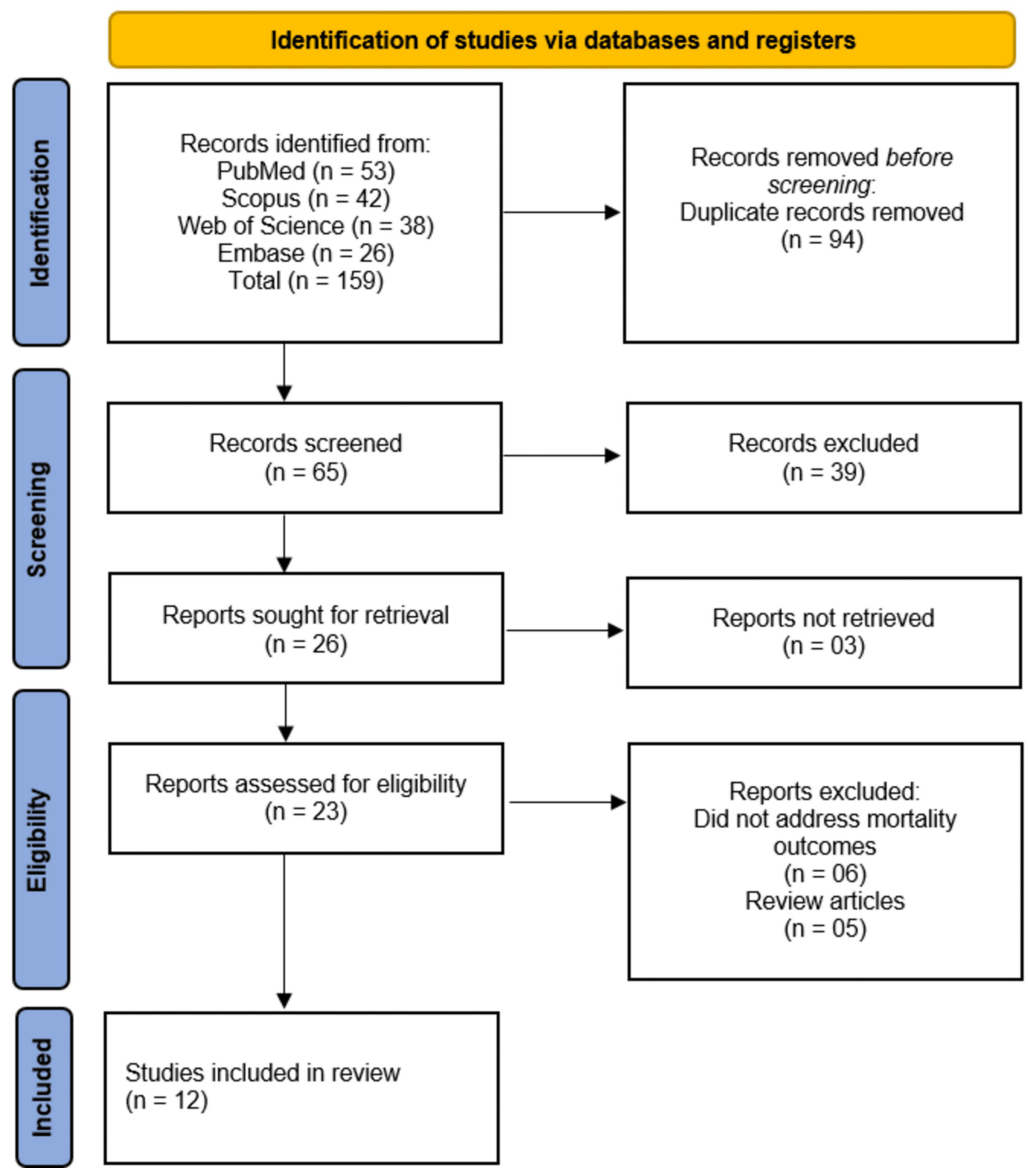
Illustration of studies selection process on PRISMA flowchart. PRISMA: Preferred Reporting Items for Systematic Reviews and Meta-Analyses.

The systematic review initially identified 159 records from PubMed (n = 53), Scopus (n = 42), Web of Science (n = 38), and Embase (n = 26). After removing 94 duplicates, 65 records were screened, of which 39 were excluded. Of the remaining 26 reports sought for retrieval, three were unavailable, leaving 23 reports assessed for eligibility. Six studies were excluded for not addressing mortality outcomes, and five were review articles. Ultimately, 12 studies [11-22] met the inclusion criteria and were included in the review (Figure 1).

Studies Characteristics

We included 12 studies [11-22] investigating the prognostic value of Gal-3 in chronic HF patients, with sample sizes ranging from 149 to 2,067 participants (Table 1). The studies were conducted across multiple countries, including the USA, France, Germany, Spain, Switzerland, Italy, the Netherlands, and Norway, reflecting a diverse patient population. Study designs varied, encompassing RCTs [11,20], prospective and retrospective cohort studies [12,14,16,17,19,21,22], and observational analyses [13,15,18]. The patient populations predominantly consisted of individuals with chronic HF, including those with reduced ejection fraction [11,15,20] and non-ischemic dilated cardiomyopathy [13]. Mean ages ranged from 56 to 71 years, with follow-up durations extending up to 8.7 years in some studies [19]. Gal-3 measurement methods included serum or plasma assays, though specific techniques were not always detailed. Cut-off values for Gal-3 varied, with some studies using quartiles [13] or median values (e.g., 13.9 ng/mL [22] or 17.8 ng/mL [16]), while others did not specify thresholds.

**Table 1:** Characteristics of included studies. RCT: randomized controlled trial; HF: heart failure; sST2; soluble suppression of tumorigenicity 2; TIMP-1: tissue inhibitor of metalloproteinase-1; CTx: C-terminal telopeptide; PIIINP: procollagen III N-terminal propeptide; DCM: dilated cardiomyopathy; ACM: all-cause mortality; CM: cardiac mortality; hs-TnT: high-sensitivity troponin T; ST2: suppression of tumorigenicity 2; TIME-CHF: Trial of Intensified versus Standard Medical Therapy in Elderly Patients with Congestive Heart Failure; GISSI-HF: Gruppo Italiano per lo Studio della Sopravvivenza nell'Insufficienza Cardiaca - Heart Failure; β-blocker: beta-blocker; RAS: renin-angiotensin system; MRA: mineralocorticoid receptor antagonist; LVEF: left ventricular ejection fraction; NT-proBNP: N-terminal pro-B-type natriuretic peptide; CHF: chronic heart failure; LV: left ventricle/left ventricular; NYHA: New York Heart Association; LVEDV: left ventricular end-diastolic volume; MI: myocardial infarction; CRP: C-reactive protein; WMSI: wall motion score index; VO₂: oxygen consumption; IQR: interquartile range; NR: not reported.

First author (year)	Country	Study design	Sample size (n)	Patient population	Mean age (years)	Follow-up duration	Galectin-3 measurement method	Galectin-3 cut-off value	Outcome(s) assessed	Key findings
Zile et al. (2019) [11]	USA	RCT	2,067 (baseline); 1,776 (baseline + 8 months)	Patients with chronic heart failure with reduced ejection fraction	NR	8 months	NR	NR	Primary composite outcome: cardiovascular death or HF hospitalization	Sacubitril/valsartan reduced multiple profibrotic biomarkers (including Gal-3) more than enalapril; higher baseline biomarker values were linked with higher outcome rates; changes in sST2 and TIMP-1 correlated with outcome change. Suggests sacubitril/valsartan reduces profibrotic signaling, contributing to better outcomes.
Dupuy et al. (2019) [12]	France	Prognostic cohort study	182	Patients with chronic heart failure	NR	NR	NR	NR	Mortality	In univariate analysis, galectin-3 predicted mortality; however, in multivariate models, galectin-3 was not a significant predictor. CTx/PIIINP ratio and sST2 remained significant predictors. A multimarker approach, including fibrosis markers, improved prognostic accuracy more than galectin-3 alone.
Binas et al. (2018) [13]	Germany	Observational cohort study	262	Patients with non-ischemic dilated cardiomyopathy (DCM), including subgroups with inflammatory/viral and idiopathic DCM	NR	NR	Serum measurement (specific assay not stated)	Quartiles used; exact cut-off not specified	All-cause mortality (ACM), cardiac mortality (CM)	Galectin-3 as a continuous variable was not significant; however, intermediate quartile values of galectin-3 were significantly associated with a lower event rate for ACM and CM.
Alonso et al. (2016) [14]	Spain	Prospective cohort	1,069	Ambulatory heart failure patients, 36% with diabetes mellitus	66.2 ± 12.8	4.9 ± 2.8 years	Serum concentration measured (exact assay not specified)	NR	All-cause mortality, cardiovascular mortality	Galectin-3 levels were higher in diabetic patients. Galectin-3 was not independently predictive of mortality in multivariable analysis—hs-TnT and ST2 were the strongest independent predictors in diabetic HF patients.
Wijk et al. (2016) [15]	Switzerland (TIME-CHF) & Italy (GISSI-HF)	Retrospective post hoc analysis of RCTs	TIME-CHF: 219; GISSI-HF: 631	Patients with heart failure and reduced ejection fraction	NR	NR	Plasma galectin-3 concentrations	NR	All-cause mortality, composite of mortality with HF hospitalization or any hospitalization	High galectin-3 levels were linked to worse prognosis in both cohorts. Low galectin-3 patients benefited more from β-blocker up-titration; high galectin-3 patients did not. Interactions were also observed with RAS blockade and spironolactone, but were less consistent.
Koukoui et al. (2015) [16]	France	Prospective cohort with propensity score matching	202 patients (101 MRA-Plus, 101 MRA-Neg)	Chronic heart failure patients	NR	3 years	Plasma galectin-3 measurement (exact assay not specified)	17.8 ng/mL	All-cause mortality	MRA treatment did not alter the prognostic value of Gal-3; Gal-3 ≥17.8 ng/mL associated with higher mortality risk regardless of MRA use.
Bayes-Genis et al. (2014) [17]	Spain	Cohort study	876	Ambulatory patients with chronic heart failure (median LVEF: 34%)	Median age: 70 years	Median: 4.2 years (5.9 for survivors)	NR	NR	5-year all-cause mortality, cardiovascular mortality, combined all-cause death/HF hospitalization	Gal-3 independently predicted mortality in bivariate analysis but did not add significant predictive value beyond conventional risk factors and NT-proBNP; ST2 was superior to Gal-3 for risk stratification.
Jungbauer et al. (2014) [18]	Germany	Observational cohort study	149 CHF patients + 84 healthy controls	Patients with chronic heart failure and healthy controls	NR	3 years	Roche Diagnostics assays	NR	All-cause mortality	Galectin-3 was significantly elevated in CHF patients, correlated with the severity of LV dysfunction and NYHA class, and predicted all-cause mortality over 3 years; a multimarker panel improved prognostic value beyond NT-proBNP alone.
Lok et al. (2013) [19]	The Netherlands	Prospective observational cohort	240	NYHA class III and IV chronic HF patients	71 ± 0.6	8.7 ± 1 years	Blood sample (exact assay not specified)	Baseline median levels compared: 14.7 vs. 17.9 vs. 19.0 ng/mL	Left ventricular remodeling (LVEDV change); long-term mortality	Higher baseline Gal-3 is associated with worse LV remodeling and higher long-term mortality; Gal-3 predicts outcome independently.
Gullestad et al. (2012) [20]	Norway	Randomized controlled trial (substudy analysis)	1,462	Patients >60 years with systolic, ischemic HF	>60	NR	NR	NR	Cardiovascular death, nonfatal myocardial infarction, stroke, all-cause mortality, cardiovascular mortality, sudden death, coronary events, HF hospitalization	Galectin-3 was initially associated with adverse outcomes, but its predictive value disappeared after adjusting for NT-proBNP, suggesting limited additional prognostic value in elderly patients with advanced systolic HF.
Ueland et al. (2011) [21]	Norway	Observational cohort study	168 HF patients; 20 healthy controls	Stable chronic HF patients (NYHA class II-IV); age and sex-matched healthy controls	56 ± 7 years	NR	Enzyme-linked immunosorbent assay (BG Medicine, Waltham, MA)	Median 15.3 ng/mL	Mortality, correlations with NT-proBNP, CRP, LVEF, WMSI, VO2	Higher galectin-3 is associated with worse NYHA class, lower VO2, higher NT-proBNP, CRP, and creatinine; galectin-3 above the median predicts higher mortality risk.
Tang et al. (2011) [22]	USA	Observational cohort study	178 total (133 chronic HF + 45 advanced decompensated HF)	Patients with chronic systolic HF and advanced decompensated HF	NR	NR	Plasma galectin-3 measured	Median 13.9 ng/ml (IQR: 12.1–16.9)	All-cause mortality; combined endpoint of mortality, cardiac transplantation, or HF hospitalization; myocardial indexes (LV function, volume)	Higher galectin-3 was associated with older age, worse renal function, and independently predicted all-cause mortality but did not predict the combined endpoint or correlate with echocardiographic/hemodynamic indices.

Prognostic Value of Galectin-3 in Mortality Outcomes

Gal-3 demonstrated mixed prognostic utility across the included studies. In univariate analyses, elevated Gal-3 levels were consistently associated with increased all-cause mortality and adverse cardiovascular outcomes. For instance, Tang et al. [22] reported a hazard ratio (HR) of 1.86 (95% CI: 1.36-2.54, p < 0.001) for all-cause mortality in chronic HF patients, while Koukoui et al. [16] found that Gal-3 ≥17.8 ng/mL was strongly predictive of mortality (HR = 7.4-9.0, p < 0.05). Similarly, Ueland et al. [21] observed that Gal-3 levels above the median (15.3 ng/mL) correlated with worse New York Heart Association (NYHA) class and higher mortality risk.

However, the independent prognostic value of Gal-3 diminished in multivariate models adjusting for established biomarkers and clinical variables. Dupuy et al. [12] found that Gal-3 lost significance when adjusted for fibrosis markers (C-terminal telopeptide (CTx)/procollagen III N-terminal propeptide (PIIINP) ratio and soluble suppression of tumorigenicity 2 (sST2)), while Alonso et al. [14] reported that Gal-3 was not independently predictive of mortality after accounting for high-sensitivity troponin T (hs-TnT) and suppression of tumorigenicity 2 (ST2). Bayes-Genis et al. [17] noted that Gal-3 added minimal incremental value beyond N-terminal pro-B-type natriuretic peptide (NT-proBNP) and conventional risk factors, with ST2 outperforming Gal-3 in risk stratification.

Interaction With Treatment Effects

Several studies explored whether Gal-3 levels influenced treatment responses. Zile et al. [11] demonstrated that sacubitril/valsartan significantly reduced Gal-3 and other profibrotic biomarkers compared to enalapril, suggesting modifiability of Gal-3 by therapy. Wijk et al. [15] reported an interaction between Gal-3 and β-blocker efficacy, with low Gal-3 patients deriving greater survival benefits from up-titration (HR: 2.42 in TIME-CHF, p = 0.02). Conversely, Koukoui et al. [16] found that mineralocorticoid receptor antagonists (MRAs) did not alter the prognostic association of elevated Gal-3 with mortality.

Comparative Performance With Other Biomarkers

Gal-3 often underperformed compared to other biomarkers in multivariable models. For example, Gullestad et al. [20] observed that Gal-3 lost predictive significance for cardiovascular outcomes after adjusting for NT-proBNP. Similarly, Binas et al. [13] reported that intermediate Gal-3 quartiles were associated with lower mortality, but continuous Gal-3 values were not significant. Jungbauer et al. [18] and Lok et al. [19] highlighted that multimarker panels incorporating Gal-3 improved prognostic accuracy beyond NT-proBNP alone, though Gal-3 alone was less robust.

The included studies collectively suggest that while Gal-3 is a promising biomarker for mortality risk stratification in chronic HF, its independent prognostic utility is limited in the context of established biomarkers like NT-proBNP, ST2, and hs-TnT (Table 2). Elevated Gal-3 levels consistently correlated with adverse outcomes, but its additive value was modest in multivariate analyses. Treatment interactions, particularly with sacubitril/valsartan and β-blockers, indicate potential therapeutic implications, though further research is needed to clarify these relationships.

**Table 2:** Statistical findings from included studies. NR: not reported; HF: heart failure; HS-cTnT: high-sensitivity cardiac troponin T; CRP: C-reactive protein; NT-proBNP: N-terminal pro-B-type natriuretic peptide; CTx: C-terminal telopeptide; PIIINP: procollagen III N-terminal propeptide; sST2: soluble suppression of tumorigenicity 2; ACM: all-cause mortality; CM: cardiac mortality; DM: diabetes mellitus; TIME-CHF: Trial of Intensified versus Standard Medical Therapy in Elderly Patients with Congestive Heart Failure; GISSI-HF: Gruppo Italiano per lo Studio della Sopravvivenza nell'Insufficienza Cardiaca - Heart Failure; HR: hazard ratio; MRA: mineralocorticoid receptor antagonist; CV: cardiovascular; CHF: chronic heart failure; LV: left ventricle/left ventricular; MI: myocardial infarction; NYHA: New York Heart Association; LVEF: left ventricular ejection fraction; IQR: interquartile range.

First author (year)	Sample size (n)	Galectin-3 cut-off value	Outcome assessed	Effect estimate	95% confidence interval (CI)	p-value	Adjusted variables	Key interpretation
Zile et al. (2019) [11]	2,067 (baseline); 1,776 (baseline + 8 months)	NR	Cardiovascular death or HF hospitalization	NR	NR	NR	NR	Galectin-3 was higher at baseline than control referents; sacubitril/valsartan significantly reduced multiple profibrotic biomarkers (including Gal-3) compared to enalapril; higher baseline profibrotic markers were associated with worse outcomes; thus, galectin-3 may have prognostic value and be modifiable by treatment.
Dupuy et al. (2019) [12]	182	NR	Mortality	NR	NR	NR	Age, clinical data, hs-cTnT, CRP, NT-proBNP, CTx/PIIINP ratio, sST2	Galectin-3 was not significantly associated with mortality in multivariate models; fibrosis markers CTx/PIIINP ratio and sST2 showed stronger prognostic value.
Binas et al. (2018) [13]	262	Quartiles (intermediate levels)	All-cause mortality (ACM) and cardiac mortality (CM)	Significant for intermediate quartiles	NR	Significant	Multivariate analysis performed; details not specified	Intermediate galectin-3 levels are associated with lower mortality events; no significant result for galectin-3 as a continuous variable.
Alonso et al. (2016) [14]	1069	NR	All-cause mortality; cardiovascular mortality	NR	NR	p < 0.001 (higher levels in DM vs. non-DM); no significant interaction with DM for mortality prediction	Multivariable Cox regression included multiple biomarkers	Galectin-3 levels were higher in diabetic HF patients, but galectin-3 was not independently predictive of all-cause or cardiovascular mortality in multivariable analysis.
Wijk et al. (2016) [15]	219 (TIME-CHF); 631 (GISSI-HF)	NR	All-cause mortality	TIME-CHF: HR 2.42; GISSI-HF: HR 1.47	TIME-CHF: 1.17–5.01; GISSI-HF: 1.02–2.10	TIME-CHF: 0.02; GISSI-HF: 0.04	Multivariate adjustment	High galectin-3 levels were significantly associated with higher mortality in both cohorts. Prognostic effect interacted with β-blocker up-titration (beneficial in low galectin-3 patients). Trends noted for RAS blockade; spironolactone effect inconsistent.
Koukoui et al. (2015) [16]	202 (101 MRA-Plus, 101 MRA-Neg)	17.8 ng/mL	All-cause mortality (3-year follow-up)	HR = 7.4 (MRA-Plus), HR = 9.0 (MRA-Neg) for Gal-3 ≥17.8 ng/mL	2.2–24.6 (MRA-Plus), 2.9–27.8 (MRA-Neg)	p = 0.539	Propensity score matching for cardiovascular risk factors; multivariate Cox model	Elevated Gal-3 (≥17.8 ng/mL) is strongly associated with higher mortality; MRA treatment did not alter this prognostic value.
Bayes-Genis et al. (2014) [17]	876	NR	All-cause mortality, cardiovascular mortality, combined all-cause death/HF hospitalization	Multivariate analysis: Gal-3 not independently associated with CV mortality; no significant increase in discrimination or reclassification	NR	NR	Adjusted for 11 clinical risk factors + NT-proBNP	Galectin-3 added minimal incremental predictive value over existing risk factors and was inferior to ST2 for risk stratification in chronic HF.
Jungbauer et al. (2014) [18]	149 patients (CHF) + 84 controls	Not specified in the abstract	All-cause mortality (3-year follow-up)	NR	NR	p < 0.05	NR	Galectin-3 was significantly elevated in CHF patients, correlated with NT-proBNP, increased with symptom severity and LV dysfunction, and predicted all-cause mortality.
Lok et al. (2013) [19]	149 patients (CHF) + 84 controls	Not specified in the abstract	All-cause mortality (3-year follow-up)	NR	NR	p < 0.05	NR	Galectin-3 was significantly elevated in CHF patients, correlated with NT-proBNP, increased with symptom severity and LV dysfunction, and predicted all-cause mortality.
Gullestad et al. (2012) [20]	1462	NR	Primary composite endpoint (CV death, nonfatal MI, stroke); all-cause mortality; CV mortality; sudden death; coronary endpoint	HR: 1.48–1.83 (varied by outcome)	1.03–2.94	0.002–0.035	Adjusted for clinical & biochemical predictors; effect attenuated after NT-proBNP added	Galectin-3 shows a significant association with mortality and cardiac outcomes before NT-proBNP adjustment, but loses significance after NT-proBNP inclusion; limited added prognostic value in older patients with advanced systolic HF.
Ueland et al. (2011) [21]	168 HF patients (plus 20 controls)	15.3 ng/mL (median)	Mortality in chronic HF	Hazard ratio implied via Cox analysis	NR	Significant if p < 0.05 (exact p not given)	NYHA class, serum creatinine, NT-proBNP, CRP, LVEF, interaction terms (galectin-3 - NT-proBNP, galectin-3 - CRP, CRP*NT-proBNP)	Galectin-3 levels above the median were associated with worse NYHA class, higher inflammation and neurohormonal markers, lower exercise capacity, and higher mortality risk.
Tang et al. (2011) [22]	133 (chronic HF) + 45 (advanced decompensated HF)	Median: 13.9 ng/ml (IQR: 12.1–16.9)	All-cause mortality (chronic HF cohort)	HR: 1.86 (univariate); HR: 1.94 (multivariate)	1.36–2.54 (univariate); 1.30–2.91 (multivariate)	p < 0.001 (univariate); p = 0.001 (multivariate)	Age, estimated glomerular filtration rate, LV ejection fraction, mitral early diastolic myocardial relaxation velocity	Higher galectin-3 independently predicts all-cause mortality in chronic HF, but not the combined endpoint (death, transplant, HF hospitalization). No relation with echocardiographic/hemodynamic indexes.

Risk of Bias Assessment Results

The risk of bias assessment using the QUIPS tool revealed that most studies had a moderate overall risk of bias, primarily due to limitations in prognostic factor measurement and adjustment for confounders. Only one study, Wijk et al. [15], was rated as low risk, owing to its robust design as an RCT subgroup analysis with clear outcome measures. Common methodological concerns included unspecified Gal-3 assay details [11-14,17,19,22] and incomplete adjustment for competing biomarkers like NT-proBNP or ST2 [12,14,20]. Studies with propensity-matched designs [16] or small sample sizes [18,21] were also prone to moderate bias. Despite these limitations, outcome measurement was consistently rated as low risk across all studies [11,22], supporting the reliability of mortality endpoint reporting (Table 3).

**Table 3:** Risk of bias results on QUIPS tool. QUIPS: Quality in Prognosis Studies.

Study (first author, year)	Study participation	Study attrition	Prognostic factor (Gal-3) measurement	Outcome measurement	Adjustment for other factors	Statistical analysis & reporting	Overall risk of bias
Zile et al. (2019) [11]	Low	Low	Moderate	Low	Moderate	Low	Moderate
Dupuy et al. (2019) [12]	Moderate	Low	Moderate	Low	High	Moderate	Moderate
Binas et al. (2018) [13]	Moderate	Low	Moderate	Low	Moderate	Low	Moderate
Alonso et al. (2016) [14]	Low	Low	Moderate	Low	High	Moderate	Moderate
Wijk et al. (2016) [15]	Low	Low	Moderate	Low	Moderate	Low	Low
Koukoui et al. (2015) [16]	Moderate	Low	Moderate	Low	Moderate	Low	Moderate
Bayes-Genis et al. (2014) [17]	Low	Low	High	Low	High	Moderate	Moderate
Jungbauer et al. (2014) [18]	Moderate	Low	Moderate	Low	Low	Low	Moderate
Lok et al. (2013) [19]	Moderate	Low	Moderate	Low	Moderate	Low	Moderate
Gullestad et al. (2012) [20]	Low	Low	High	Low	High	Moderate	Moderate
Ueland et al. (2011) [21]	Moderate	Low	Low	Low	Moderate	Low	Moderate
Tang et al. (2011) [22]	Moderate	Low	Moderate	Low	Moderate	Low	Moderate

Discussion

The findings of this systematic review highlight the complex and multifaceted role of Gal-3 as a prognostic biomarker in chronic HF. Across the 12 included studies, Gal-3 consistently demonstrated an association with adverse mortality outcomes in univariate analyses, reinforcing its potential as a marker of disease severity and progression. For instance, Tang et al. [22] reported a significant HR of 1.86 (95% CI: 1.36-2.54, p < 0.001) for all-cause mortality, while Koukoui et al. [16] identified a strong predictive value for Gal-3 levels ≥17.8 ng/mL (HR = 7.4-9.0, p < 0.05). These results align with existing literature, such as the work of de Boer et al. [23], who similarly found that elevated Gal-3 levels were linked to increased mortality in HF patients, particularly in those with preserved ejection fraction. However, the robustness of Gal-3 as an independent prognostic marker diminishes when contextualized within multivariable models that account for established biomarkers like NT-proBNP, ST2, and hs-TnT. This was evident in studies such as Dupuy et al. [12] and Alonso et al. [14], where Gal-3 lost statistical significance after adjustment for competing risk factors, suggesting that its predictive value may be secondary to more potent biomarkers or confounded by underlying pathophysiological processes.

The variability in Gal-3’s prognostic performance across studies may be attributed to differences in study design, patient populations, and methodological approaches. For example, the study by Wijk et al. [15], which derived data from RCT subgroups (TIME-CHF and GISSI-HF), reported a significant interaction between Gal-3 levels and β-blocker efficacy, with low Gal-3 patients benefiting more from up-titration (HR: 2.42, p = 0.02). This finding suggests that Gal-3 might serve as a modifier of treatment response, a hypothesis supported by prior research, such as the PROTECT trial [24], which identified Gal-3 as a potential mediator of therapeutic effects in HF. Conversely, the study by Koukoui et al. [16] found that MRAs did not alter the prognostic association of Gal-3 with mortality, indicating that the biomarker’s predictive utility may be treatment-invariant in certain contexts. Furthermore, recent studies have demonstrated a positive correlation between Gal-3 levels and myocardial fibrosis quantified by late gadolinium enhancement (LGE) on cardiac magnetic resonance imaging, supporting its role as a marker of cardiac fibrosis burden. This imaging-based correlation reinforces the mechanistic link between elevated Gal-3, increased fibrosis, and adverse outcomes in HF. These discrepancies underscore the need for further investigation into the mechanisms by which Gal-3 interacts with HF therapies, particularly in light of its role in fibrosis and inflammation, as highlighted by Henderson et al. [25].

One of the most striking observations from this review is the comparative inferiority of Gal-3 to other biomarkers, such as ST2 and NT-proBNP, in multivariable models. Bayes-Genis et al. [17] directly compared Gal-3 and ST2, concluding that ST2 provided superior risk stratification and incremental prognostic value. This aligns with findings from the MADIT-CHIC trial [26], which demonstrated ST2’s robustness in predicting arrhythmic events and mortality in HF. Similarly, Gullestad et al. [20] observed that Gal-3’s predictive significance for cardiovascular outcomes disappeared after adjusting for NT-proBNP, echoing results from the CORONA trial [27], where NT-proBNP consistently outperformed Gal-3 in risk prediction. These comparisons suggest that while Gal-3 may contribute to risk assessment, its clinical utility is likely greatest when integrated into multimarker panels, as proposed by Jungbauer et al. [18] and Lok et al. [19]. Such panels could leverage the complementary strengths of Gal-3 (reflecting fibrosis and inflammation) and NT-proBNP (reflecting hemodynamic stress) to provide a more comprehensive prognostic picture.

The modifiability of Gal-3 by therapeutic interventions represents another critical dimension of its clinical relevance. Zile et al. [11] demonstrated that sacubitril/valsartan significantly reduced Gal-3 levels compared to enalapril, suggesting that Gal-3 may be a dynamic marker responsive to treatment. This finding is supported by experimental studies, such as those by Salloum et al. [28], which showed that Gal-3 inhibition attenuates cardiac fibrosis in animal models. However, the clinical implications of these observations remain uncertain, as reductions in Gal-3 did not uniformly translate to improved outcomes in all studies. For instance, while Wijk et al. [15] reported treatment interactions with β-blockers, the effects were inconsistent across cohorts, and Koukoui et al. [16] found no modulation of Gal-3’s prognostic value by MRAs. These mixed results mirror broader debates in the literature about the causal role of Gal-3 in HF progression. Some researchers, like Sharma et al. [29], argue that Gal-3 is merely a bystander biomarker, while others, such as Meijers et al. [30], posit that it actively contributes to myocardial remodeling. Resolving this question will require mechanistic studies and randomized trials targeting Gal-3 directly, akin to ongoing investigations into anti-fibrotic therapies.

The heterogeneity in Gal-3 measurement methods and cut-off values across studies further complicates its clinical application. While some studies, like Ueland et al. [21], used standardized enzyme-linked immunosorbent assay (ELISA) assays, others did not report assay details [11,12], raising concerns about reproducibility. This issue is not unique to Gal-3; similar challenges have been noted for other emerging biomarkers, as discussed by Ky et al. [31]. Standardization efforts, such as those led by the FDA’s Biomarker Working Group [32], could mitigate these discrepancies and enhance the comparability of future research. Additionally, the lack of consensus on optimal Gal-3 cut-off values, ranging from 13.9 ng/mL [22] to 17.8 ng/mL [16], limits its readiness for clinical implementation. This variability contrasts with established biomarkers like NT-proBNP, for which universally accepted thresholds exist [33], and underscores the need for large-scale validation studies to define clinically meaningful Gal-3 thresholds.

The patient populations examined in the included studies also varied widely, from non-ischemic dilated cardiomyopathy [13] to advanced decompensated HF [22], potentially influencing Gal-3’s prognostic performance. For example, Alonso et al. [14] focused on diabetic HF patients, finding that Gal-3 levels were higher in this subgroup but lacked independent predictive value. This aligns with research by Bošnjak et al. [34], which highlighted the confounding effects of comorbidities like diabetes on biomarker performance. Similarly, the study by Binas et al. [13] reported that intermediate Gal-3 quartiles, rather than continuous values, were predictive in non-ischemic HF, suggesting that Gal-3’s utility may be context-dependent. These findings emphasize the importance of tailoring biomarker use to specific HF phenotypes, as advocated in the ESC guidelines [35].

Limitations

This systematic review has several limitations. First, the included studies exhibited methodological heterogeneity, particularly in Gal-3 measurement techniques and statistical adjustments, which may have influenced the consistency of findings. Second, the predominance of observational designs limits causal inferences about Gal-3’s role in HF progression. Third, publication bias cannot be ruled out, as negative studies may have been underrepresented. Finally, the lack of individual patient data precluded subgroup analyses by HF phenotype or treatment regimen.

## Conclusions

While elevated Gal-3 levels consistently correlate with adverse outcomes, its independent predictive value is often eclipsed by established biomarkers like NT-proBNP and ST2. The biomarker’s responsiveness to certain therapies, such as sacubitril/valsartan, and its interactions with treatment effects, as seen with β-blockers, suggest that Gal-3 may have a role in personalized HF management. However, standardization of assays, validation of cut-off values, and further mechanistic research are needed to clarify its clinical utility. Until then, Gal-3 is best viewed as a complementary tool within multimarker strategies, rather than a standalone prognostic indicator. Future studies should prioritize randomized controlled trials to evaluate whether targeting Gal-3 directly improves outcomes, thereby bridging the gap between biomarker research and therapeutic innovation.

## Appendices

**Table 4:** Search strategy.

Database	Search string
PubMed	("Galectin 3"[Mesh] OR "Galectin-3" OR "Gal-3") AND ("Heart Failure"[Mesh] OR "heart failure" OR "cardiac failure" OR "congestive heart failure" OR "CHF") AND ("Mortality"[Mesh] OR mortality OR death OR survival)
Scopus	TITLE-ABS-KEY ("Galectin-3" OR "Gal-3") AND TITLE-ABS-KEY ("heart failure" OR "cardiac failure" OR "congestive heart failure" OR "CHF") AND TITLE-ABS-KEY (mortality OR death OR survival)
Web of Science	TS=("Galectin-3" OR "Gal-3") AND TS=("heart failure" OR "cardiac failure" OR "congestive heart failure" OR "CHF") AND TS=(mortality OR death OR survival)
Embase	('galectin 3'/exp OR 'galectin-3' OR 'gal-3') AND ('heart failure'/exp OR 'heart failure' OR 'cardiac failure' OR 'congestive heart failure' OR 'CHF') AND ('mortality'/exp OR mortality OR death OR survival)
